# The Effect of Art Therapy in Women with Gynecologic Cancer: A Systematic Review

**DOI:** 10.1155/2020/8063172

**Published:** 2020-04-24

**Authors:** Wenjing Fu, Yan Huang, Xing Liu, Jianhua Ren, Mengqin Zhang

**Affiliations:** ^1^Nursing Department, West China Second University Hospital, Sichuan University, Chengdu, Sichuan 610041, China; ^2^Key Laboratory of Birth Defects and Related Diseases of Women and Children, Sichuan University, Ministry of Education, Chengdu, Sichuan 610041, China

## Abstract

**Objectives:**

The purpose of this study was to evaluate the evidence of art therapy on the psychological outcome, quality of life (QOL), and cancer-related symptoms in women with gynecological cancer.

**Methods:**

A systematic literature search was conducted. The randomized controlled trials, quasiexperimental studies, case reports, and qualitative studies were all included.

**Result:**

1,587 articles were retrieved. A total of 9 articles met the inclusion criteria. The existing studies provided initial evidence to suggest that art therapy may benefit gynecological cancer patients with respect to improving psychological outcome and QOL, reducing fatigue related to cancer, and improving subjective overall health condition. However, the quality of the current evidence limits the efficacy of these findings.

**Conclusion:**

Research on art therapy of gynecologic cancer patients is insufficient. We cannot draw the conclusion that art therapy benefits gynecological cancer patients in the psychological outcome, QOL, and cancer-related symptoms. More rigorous research is needed.

## 1. Introduction

Cancer is an overwhelming experience for patients [1]. The diagnosis of cancer has not only obvious physical ramifications but also produces psychological stress as well [2]. Cancer patients are particularly vulnerable to depression and anxiety. These disorders have a severely detrimental effect on the patient's quality of life [3]. Gynecologic cancers include any cancer of the female genital system: uterine, ovarian, cervical, vulvar, vaginal, and endometrial [2]. These diseases seriously affect women's health. For example, cervical cancer is the fourth most common cancer in women, and the seventh overall, accounting for 7.5% of all female cancer deaths [4]. The patients with gynecological cancer may suffer from psychological distress, fatigue, sleep disturbance, urinary/bowel issues, lymphedema, menopausal symptoms, and sexuality issues [5–7]. Side effects of treatment, complications, and cumulative organ toxicities may alter the patient's body function, interfere with daily activities, have a negative impact on QOL [8], as well as distorting their psychological states, body image, body awareness, and self-esteem [2]. It was reported that nearly 90% of gynecological cancer survivors need supportive care [9]. Cancer patients are increasingly turning to complementary and alternative medicine (CAM) therapies to reduce symptoms, improve QOL, and boost their ability to cope with stress [10]. Art therapy is one of the complementary therapies being used to relieve cancer symptoms [11].

Art therapy is an umbrella term for therapies such as dance and movement therapy, music therapy, and art therapy working with visual arts materials. It is a clinical intervention based on the belief that the creative process involved in the making of art is healing and life enhancing [11]. Art therapy can provide a route of nonverbal expression; the process of creating art provides relief and support. The majority of art therapy research has involved patients with breast cancer, hematologic malignancies, and pediatric brain tumors [12]. Studies have provided encouraging evidence to utilize this technique. It has become increasingly popular in a number of medical and health fields [10]. Cancer patients participating in art therapy experience improvements in mental health, QOL, personal growth, or positive social interaction [13]. Studies have suggested that art therapy leads to increased awareness of self, improved ability to cope with symptoms, stress, and traumatic experiences [14].

Some systematic reviews have explored the effects of art therapy. For example, Tang et al. [1] reported that art therapy benefited female breast cancer patients with respect to relieving anxiety, depression, and fatigue. Wood et al. [15] found a positive effect of art therapy on psychological symptoms and QOL in adults with cancer. A review by Geue et al. [13] showed that art therapy benefited cancer patients in various ways, including improving their mental health. The review of Reynolds et al. [16] showed that three types of study designs produced similar results regarding the positive effects of art therapy, but inconsistency was found. A systematic review [10] suggested that arts therapies seemed to positively affect the anxiety of cancer patients but not depression or QOL. Besides, patients with different cancer types are heterogeneous in terms of sociodemographic factors, symptoms, treatment, and side effects [10]. Considering the characteristics of the disease and its impact on body image, fertility, and sexuality, art therapy focusing on gynecological cancer patients need to be explored specifically. To our knowledge, no review has specifically focused on this field. This systematic review aims to evaluate the evidence for the effects of art therapy on the psychological outcome, QOL, and cancer-related symptoms in gynecological cancer patients.

## 2. Material and Methods

### 2.1. Search Strategy

A systematic approach based on the PRISMA guidelines [17] was implemented. The keywords art therapy (exp ART THERAPY/OR exp ART THERAPISTS/) AND ((exp Genital Neoplasms, Female/) or (exp Fallopian Tube Neopla/) or (exp Ovarian Neoplasms/) or (exp Brenner Tumor/) or (Carcinoma, Endometrioid/) or (exp Luteoma/) or (Carcinoma, Ovarian Epithelial/) or (exp Granulosa Cell Tumor/) or (exp Meigs Syndrome/) or (exp Sertoli-Leydig Cell Tumor/) or (exp Thecoma/) or (exp Uterine Neoplasms/) or (exp Endometrial Neoplasms/) or (exp Uterine Cervical Neoplasms/) or (exp Vaginal Neoplasms/) or (exp Vulvar Neoplasms/) were searched in the following databases: Medline, Embase, Cochrane Central Register of Controlled Trials (CCRCT), China National Knowledge Infrastructure (CNKI), Chinese Biomedical Literature Database (CBM), and Wan Fang Data. There was no time and language restriction. Both computerized and manual search methods were used. The retrieval time was 2019-4-30.

### 2.2. Inclusion and Exclusion Criteria

Participants were adult women (18 years or older) diagnosed with gynecological cancer; Art therapy intervention; Outcomes included psychological outcome, QOL, and cancer-related symptoms. Randomized controlled trials, quasiexperimental studies, case reports, and qualitative studies were included in this review. Exclusions criteria: patients with psychosis; samples composed of pediatric patients.

### 2.3. Assessment of Quality

Two authors reviewed each type of study with the JBI Critical Appraisal Checklist [18]. Disagreements were resolved through discussion with the third author.

### 2.4. Data Extraction and Analysis

Two reviewers independently extracted the required data, including participant characteristics, study methods, interventions, and outcomes. Randomized controlled trials included in the review were pooled in Meta-analyses using RevMan5.3 software program. Randomized controlled trials, quasiexperimental studies, case reports, and qualitative studies were discussed in descriptive comparisons.

## 3. Result

### 3.1. Study Identification

**Figure 1:**
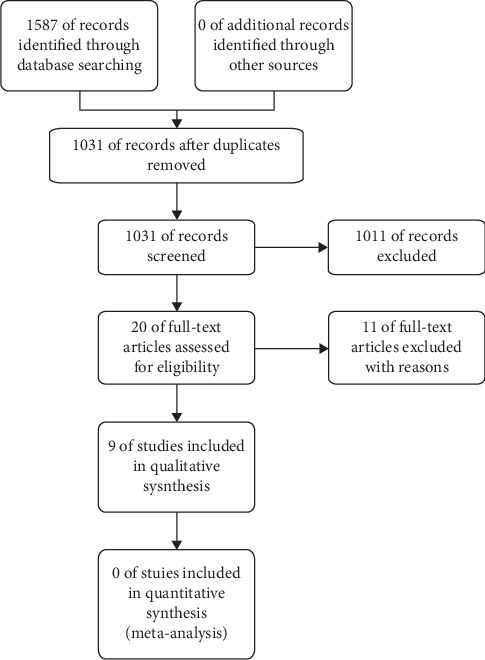
Retrieval strategy.

Our research identified 1587 items, of which 20 were relevant according to the title and abstract after duplicates were removed. 12 items were excluded based on the inclusion or exclusion criteria. 9 studies were eligible for the final review (Figure 1). The included studies ranged from 2012 to 2019, including 2 RCTs, 2 quasirandomized trial, 2 qualitative research studies, and 3 case reports. General characteristics of the included studies were listed in Table 1.

### 3.2. Participant Characteristics

The sample sizes ranged from 1 to 27. The participants of three studies [2, 19, 20] were patients receiving radiotherapy. Four studies [12, 21–23] focused on patients being treated with chemotherapy. The participants of one study [24] were newly diagnosed with gynecologic cancer patients. One eligible study [25] focused on patients who had been diagnosed for the first time with cancer and had received surgical treatment. The participants of four studies [19–21, 25] included other cancer patients than gynecological cancers. The participant characteristics were summarized in Table 1.

### 3.3. Intervention

There was considerable heterogeneity in the study designs. The art interventions included music therapy, painting appreciation, drawing, creative artwork generation, printmaking, mandala, collage, storytelling combined with dancing and crafts, writing poems, and composing tone. The length of intervention sessions varied from 30 to 90 minutes, and sessions varied from a single to an average of ten. Six papers reported that art interventions were delivered by a therapist, and one paper reported the study was supported by a therapist. However, some papers did not report whether the therapist was involved in the research. Specific information including the length and frequency of each intervention was not described clearly either.

### 3.4. Outcome

Alcântara-Silva et al. [20] found that individual music therapy sessions might be effective in reducing symptoms of depression and fatigue related to cancer, as well as to improve QOL for women with gynecological cancer undergoing radiotherapy. Lee et al. [21] identified that listening to recorded monochord sounds had positive effects on reducing anxiety and improving physical and psychological states during chemotherapy. According to Wiswell et al. [12], art therapy, consisting of drawing, printmaking, mandala, and collage, might help to prevent or mitigate the decline of QOL caused by chemotherapy in gynecological cancer patients. Lee et al. [19] found that art therapy based on famous painting appreciation and creative artwork significantly improved cancer-related anxiety and depression. The qualitative research conducted by Hammer et al. [24] indicated that drawing and later interviews might be a tool in understanding the hope of newly diagnosed gynecologic oncological patients. The research of La Cour et al. [25] demonstrated that the use of storytelling intertwined with dancing, arts, and crafts, provided the patients and their relatives with strategies to manage cancer-related concerns. Researchers [2] developed a clinical model of dance/movement therapy for gynecologic cancer patients. They did not research the efficacy in relation to clinical outcome variables, but evidence indicated the potential benefits of art therapy. The patient was able to gain insight into parts of herself and expressed being less isolated as well as more open to the support available from others. Findings of a research conducted by Ben-Arye et al. [23] suggested that music therapy may alleviate adverse symptoms of chemotherapy and improve patients' QOL. Lee et al. [22] found music therapy helped the patient to experience a decrease of negative feelings, an increase of the positive feelings, and improvement of subjective overall health condition.

### 3.5. Risk of Bias and Quality Assessment

We assessed the methodological quality of the studies and the possibility of bias with JBI Critical Appraisal Tools. The nine eligible studies varied in design, requiring different quality appraisal methods. The risk of bias and quality assessment of two RCTs is showed in Table 2.  Table 3 shows the risk of bias and quality assessment of two quasiexperiments. The assessment of two qualitative research studies is listed in Table 4.  Table 5 shows the assessment of three case reports.

## 4. Discussion

A total of nine studies were included in this review. Obvious heterogeneity of the eligible studies was observed, including research design, specific intervention method, and outcomes evaluation. For example, some studies used a single type of art intervention, while others included several types of arts. Interventions were implemented in different ways including individual or group formats. One study [25] involved the patient's relative to join, while other studies did not. The heterogeneity made it difficult to assess the studies quantitatively and generalize the findings. Consequently, the studies were qualitatively described. No overall effect size was determined. According to the current research evidence, positive results of art therapy were found. It showed that art therapy benefited gynecologic cancer patients with respect to psychological outcomes, such as anxiety, depression, and cancer-related concerns [19–22, 25]. A positive effect on QOL was also observed [12, 20, 23]. In addition, there was evidence suggesting that art therapy was helpful to reduce fatigue related to cancer [20] and improve subjective overall health condition [22].

However, the methodological shortcomings limited the efficacy of these findings. For example, the sample size of eligible studies was small. The a priori power calculation for an adequate sample size was unclear. Among them, only one quasiexperiment study, one qualitative study, and three case studies especially focused on gynecologic cancer patients, while other studies included not only gynecological patients but also other cancer patients. Lack of high-quality randomized controlled study and the absence of intent-to-treat analyses also prevented the efficacy of the findings. In addition, art therapist is someone who has a large territory of knowledge including counseling, marriage and family therapy, art, education, community work, and creative expression and integrates these fields into a unique profession [26]. The way art therapy is perceived responses to the way it is conveyed [26]. However, six of the included studies reported that art intervention was delivered by the therapist, and one research was supported by the therapist. In two research studies [24, 25], it was unclear whether the therapist was involved in the study or the implementers had received professional art therapy training. In addition, surgery is a primary treatment for gynecologic malignancy [27]. For example, hysterectomy is one of the most common gynecological surgeries. Women undergoing this surgery often experience negative emotions. It may be a potent stimulus for stress and psychological problems [28]. In the present review, we noticed that most of the participants were receiving chemotherapy or radiotherapy patients. Evidence of art therapy in gynecological perioperative cancer patients was quite insufficient.

Art therapy effectiveness research, including experimental and control group studies, is relatively new [26]. Considering the quality of current evidence, we find that research on art therapy of gynecologic cancer patients is insufficient. We cannot draw the conclusion that art therapy benefits gynecologic cancer patients in the psychological outcome, QOL, and cancer-related symptoms. There is much to be considered as to promoting art therapy in a medical setting, such as the patient's ability to move, level of verbal communication, disruptions by medical treatment, as well as the limitation of the medical environment [26]. Better integration of art therapy and medical activity to benefit gynecologic cancer patients needs to be explored further. Future research may target adequate samples, various art forms, and long-term follow-up to develop appropriate art interventions for gynecologic cancer patients receiving different treatments. Additionally, detailed sociodemographic and medical information should be taken into consideration; the mediator and moderator variables may be explored further.

## Figures and Tables

**Table 1:** Study characteristics.

Author (date)	Design	Participants (*N*)	Intervention	Comparison	Outcomes	Results
Alcântara-Silva et al. (2018)	RCT	Radiotherapy cancer patients (116), including 27 gynecological patients	30 to 40 minutes sessions of music therapy implemented by the therapist during the first week of radiotherapy, on the week of the intermediary phase, and during the last week of radiotherapy. Twice a week in the hospital	Ordinary radiotherapy	Fatigue (FACT-F); quality of life (FACT-G); depression (BDI)	FACT-F results were significant regarding trial outcome index (*P*=0.011), FACT-G (*P*=0.005), and FACT-F (*P*=0.001) for the music therapy group compared with the control group

Lee et al. (2012)	RCT	Cancer patients who were about to receive chemotherapy for the first time (40), including 2 gynecological patients	25 min of monochord (MC) sounds was used as music therapy. There was a verbal introduction (4 min) before and a silent period (5 min) after each treatment, producing a total listening time of 34 min, from the second to the fifth sessions of chemotherapy. Research supported by the therapist	Progressive muscle relaxation (PMR)	Each session was investigated before and after using SAI and a questionnaire about the patient's physical and psychological states. For the first and the last sessions, multivariate electroencephalogram (EEG) signals were recorded.	Both groups showed significantly reduced anxiety and improved physical and psychological state. EEG data demonstrated that both treatments were associated with an increase of posterior theta band activity and a decrease of midfrontal beta band activity

Wiswell et al. (2019)	Quasiexperiment study	Patients with gynecologic cancer receiving chemotherapy (16)	Five sessions of art therapy, consisting of drawing, printmaking, mandala, collage, during the chemotherapy were delivered by an art therapist. Each session was approximately 40–50 min	No	Quality of life (FACT-G)	The mean FACT-G score before chemotherapy was 82.3, and after art therapy was 78.6. The mean change in QOL was −3.7 points. A supplemental questionnaire indicated that 15 of 16 patients felt that art therapy was beneficial at each session

Lee et al. (2017)	Quasiexperiment study	Radiotherapy cancer patients (20), including cervix cancer patient (1)	The art therapy took place in two parts comprising 4 sessions of famous painting appreciation and 4 sessions of creative artwork generation; these 30 min sessions were performed by art therapist twice weekly over four weeks	No	Cancer-related distress (HADS, HDRS, ESAS)	Significant improvements in HADS anxiety and total scores were observed. HDRS scores demonstrated significant decreases. Fewer patients met the HADS or HDRS criteria for severe anxiety or depression after the intervention. No changes in ESAS mean scores were observed

Hammer et al. (2013)	Qualitative research	Newly diagnosed with gynecologic oncological patients (15)	Drawings. It was not mentioned whether a therapist was involved	No	Analyze the drawings and interview texts	Inner willpower, experiences in open nature, and closeness to loved ones contribute to hope when newly diagnosed with gynecologic cancer. Drawings and later interviews give a new understanding of the experience of hope

La cour et al. (2016)	Qualitative research	Cancer patients (10) who had received surgical treatment, including gynecological patients (8) with their relatives	Storytelling combined with dancing and crafts was a part of a rehabilitation course conducted twice by counselors	No	Cancer-related concerns. Data were generated through ethnographic fieldwork, including observations, conversations, and interviews. Participant observations and conversations were carried out during the course. Interviews were conducted one month later	It provided the patients and their relatives with strategies to manage cancer-related concerns

Ginsburgs and Goodill (2009)	Case report	Ovarian cancer patient had a hysterectomy and was currently receiving chemotherapy, being treated with radiotherapy (1)	A ten-week dance/movement therapy implemented by the therapist. Sessions were held on a weekly basis for ninety minutes, in a closed group format		The efficacy of clinical outcome variables was not researched.	Only anecdotal evidence suggested the potential benefits of the intervention. The patient was able to gain insight into parts of herself that she had suppressed during her treatments. She expressed less isolated as well as more open to the support available from others. She hoped to join the group again

Ben-Arye et al. (2015)	Case report	Recurrent ovarian cancer patient following extensive gynecological surgery, receiving chemotherapy (1)	Weekly music therapy, including Listening to individually tailored music, writing for her own poem and composing tones, conducted by the music therapist along 6 chemotherapy cycles	No	Quality of life: alleviate nausea, fatigue, and pain, relieve anxiety and depression	The patient reported significant improvement of the symptoms that impaired her QOL

Lee et al. (2015)	Case report	Ovarian cancer patient had a total hysterectomy, receiving chemotherapy (1)	Four sessions of oriental medicine music therapy based on the theories of traditional Korean medicine, twice per week for 2 weeks, for about 1 hour each time, implemented by the therapist	No	Negative and positive feelings (A self-administered questionnaire); subjective health condition (VAS)	Negative feelings decreased from a score of 17 to 7 (41% improvement); positive feelings increased from a score of 3 to 14 (more than a quadruple improvement); the VAS score increased from 40 mm to 70 mm (75% improvement)

Outcome: FACT-F: functional assessment of cancer therapy fatigue; FACT-G: functional assessment of cancer therapy-general; BDI: Beck Depression Inventory; SAI: state anxiety inventory; HADS: hospital anxiety and depression scale; HDRS: Hamilton depression rating scale; ESAS: Edmonton symptom assessment scale; VAS: visual analog scale.

**Table 2:** Risk of bias and quality assessment of RCT.

Item	Yes/no/unclear/not applicable	
	Alcântara-Silva et al. (2018)	Lee et al. (2012)
1. Was true randomization used for the assignment of participants to treatment groups?	Unclear	Yes
2. Was allocation to treatment groups concealed?	Yes	Unclear
3. Were treatment groups similar at the baseline?	Yes	Yes
4. Were participants blind to treatment assignment?	No	No
5. Were those delivering treatment blind to treatment assignment?	No	No
6. Were outcomes assessors blind to treatment assignment?	Yes	Yes
7. Were treatment groups treated identically other than the intervention of interest?	Unclear	Unclear
8. Was follow-up complete and if not, were differences between groups in terms of their follow-up adequately described and analyzed?	Unclear	Unclear
9. Were participants analyzed in the groups to which they were randomized?	Unclear	Unclear
10. Were outcomes measured in the same way for treatment groups?	Yes	Yes
11. Were outcomes measured in a reliable way?	Yes	Yes
12. Was an appropriate statistical analysis used?	Yes	Yes
13. Was the trial design appropriate, and any deviations from the standard RCT design (individual randomization, parallel groups) accounted for in the conduct and analysis of the trial?	Unclear	Unclear

**Table 3:** Risk of bias and quality assessment of quasiexperiment study.

Item	Yes/no/unclear/not applicable	
	Wiswell et al. (2019)	Lee et al. (2017)
1. Is it clear in the study what is the “cause” and what is the “effect” (i.e., there is no confusion about which variable comes first)?	Yes	Yes
2. Were the participants included in any comparisons similar?	Not applicable	Not applicable
3. Were the participants included in any comparisons receiving similar treatment/care, other than the exposure or intervention of interest?	Not applicable	Not applicable
4. Was there a control group?	No	No
5. Were there multiple measurements of the outcome, both before and after the intervention/exposure?	Yes	Yes
6. Was follow-up complete and if not, were differences between groups in terms of their follow-up adequately described and analyzed?	Yes	Yes
7. Were the outcomes of participants included in any comparisons measured in the same way?	Not applicable	Not applicable
8. Were outcomes measured in a reliable way?	Yes	Yes
9. Was an appropriate statistical analysis used?	Yes	Yes

**Table 4:** Risk of bias and quality assessment of the qualitative study.

Item	Yes/no/unclear/not applicable	
	Hammer et al. (2013)	La Cour et al. (2016)
1. Is there congruity between the stated philosophical perspective and the research methodology?	Yes	Yes
2. Is there congruity between the research methodology and the research question or objectives?	Yes	Yes
3. Is there congruity between the research methodology and the methods used to collect data?	Yes	Yes
4. Is there congruity between the research methodology and the representation and analysis of data?	Yes	Yes
5. Is there congruity between the research methodology and the interpretation of results?	Yes	Yes
6. Is there a statement locating the researcher culturally or theoretically?	No	No
7. Is the influence of the researcher on the research, and vice versa, addressed?	Yes	Yes
8. Are participants, and their voices, adequately represented?	Yes	Yes
9. Is the research ethical according to current criteria or, for recent studies, and is there evidence of ethical approval by an appropriate body?	Yes	Yes
10. Do the conclusions drawn in the research report flow from the analysis, or interpretation, of the data?	Yes	Yes

**Table 5:** Risk of bias and quality assessment of the case report.

Item	Yes/no/unclear/not applicable		
	Ginsburgs and Goodill (2009)	Ben-Arye et al. (2015)	Lee et al. (2015)
1. Were patient's demographic characteristics clearly described?	Yes	Yes	Yes
2. Was the patient's history clearly described and presented as a timeline?	Yes	Yes	Yes
3. Was the current clinical condition of the patient on the presentation clear described?	Yes	Yes	Yes
4. Were diagnostic tests or assessment methods and the results clearly described?	No	Yes	Yes
5. Was the intervention(s) or treatment procedure(s) clearly described?	Yes	Yes	Yes
6. Was the postintervention clinical condition clearly described?	Yes	Yes	Yes
7. Were adverse events (harms) or unanticipated events identified and described?	No	No	No
8. Does the case report provide takeaway lessons?	Yes	Yes	Yes
